# Foreign Bodies in the Oesophagus: The Experience of the Buenos Aires Paediatric ORL Clinic

**DOI:** 10.1155/2010/490691

**Published:** 2010-09-20

**Authors:** Alberto Chinski, Francesca Foltran, Dario Gregori, Simonetta Ballali, Desiderio Passali, Luisa Bellussi

**Affiliations:** ^1^Faculty of Medicine, University of Buenos Aires, C1121ABG Buenos Aires, Argentina; ^2^Department of Surgery, University of Pisa, 56126 Pisa, Italy; ^3^Labs of Epidemiological Methods and Biostatistics, Department of Environmental Medicine and Public Health, University of Padova, Via Loredan 18, 35131 Padova, Italy; ^4^Faculty of Medicine, University of Padova, 35122 Padova, Italy; ^5^ENT Department, University of Siena, 53100 Siena, Italy

## Abstract

The ingestion of foreign bodies causing esophageal injuries is a common event, mostly in children's population. The aim of the present paper is to present foreign body (FB) ingestion cases observed in a five-year period at the Children's Hospital Gutierrez, Buenos Aires, Argentina and to compare the main findings with data coming from other well-known case series, already published in scientific literature. A prospective study on 320 of esophageal foreign body was carried out , with regard to age and sex distributions, type, dimensions and consistency, location, clinical presentation, removal and complications. In the majority of cases injuries happened while children were playing and in 85.3% adults were present. Children most frequently ingested coins (83.8% cases). Removal was performed in all cases under general anaesthesia, in 34 by esophageal forceps and in 286 cases by Magill hypopharyngeal forceps. Just one case showed complications, presenting esophageal perforation. The final results of this study show that injuries usually happen under adults' supervision and highlight that FBs involved in the incident belong to classes of objects not conceived for children's use and not suitable for their age. Therefore, educational strategies regarding safe behaviours have a key role in FB injuries prevention.

## 1. Introduction

Foreign body (FB) ingestion is a frequent occurrence in children, especially in their first six years of life [1, 2], with a peak in children older than 3 years [3, 4]. Various reasons for this event can be pointed out, stressing that all the characteristics such as sex, age, socioeconomic level and parents' influence are closely interrelated [5]. The most common aspects presented in literature as leading factors to those injuries include children's behaviour, anatomical characteristics, and physiological features such as immature swallowing coordination, development of chewing capacity, and higher respiratory rates [6]. Details of FB characteristics and the dynamics of the traumatic events involved in FB inhalation are therefore important to understand the pathogenic pathway.

The aim of the present paper is to present esophageal foreign bodies cases observed at the Children's Hospital Gutierrez in Buenos Aires in a period of five years and to compare these findings with data coming from representative case series already published in international literature and coming from other continents.

## 2. Methods

### 2.1. Data Collection

Data regarding children (0–14 years) presenting FB in the esophagus have been prospectively collected at the Children's Hospital Gutierrez in Buenos Aires over a five-year period.

### 2.2. Statistical Analysis

Details on injuries were collected, and a descriptive statistical analysis regarding (i) children demographic characteristics (age and sex), (ii) features of the object (dimensions and consistency), (iii) circumstances of the injury, (iv) clinical presentation (v) FB locations, and (vi) outcomes (complications and removal's strategy) was provided. 

Moreover, a search on PubMed database has been performed in order to retrieve other case series describing FB in the esophagus, representative of different cultural and geographical backgrounds. In 2009, Gregori et al. collected data on FB injuries in the aerodigestive tract in paediatric patients from 19 European Hospitals, recording 2103 injuries occurring in the years 2000–2002 [7]. In 2007, Lin et al. performed a study on foreign body ingestion over a 5-year period in children living in Taiwan, reviewing medical records of children who were referred to the paediatric emergency department of a single tertiary referral centre between December 2001 and May 2006 [8]. A total of 74 patients underwent an endoscopic procedure because of suspected foreign body ingestion, and in 38 cases the object was located in the esophagus. In 2006, Little et al. conducted a retrospective analysis of the medical records of children who were referred to Children's Mercy Hospital, Kansas City, Missouri, with a foreign body lodged in the esophagus [9]. Over the 16-year period, from January 1988 to October 2004, 555 children had that discharging diagnosis. In 2003, Van As et al. analyzed injuries due to FB ingestion among the 88822 patient streated in their trauma unit from 1991 to 2000. Among those injuries, 753 were FBs wedged in the esophagus [10].

From all revised papers, data regarding children age and sex, FB type, location, adult supervision, most frequent symptom and complication, and delay at diagnosis, were drawn and compared with our experience.

## 3. Results

During the five-year study period, 320 cases of foreign body ingestion with esophageal location were observed. Children's mean age was 4.58 years (SD 1.42), ranging from 2 to 9 years old. In the majority of cases (304 patients), injuries happened while the child was playing; in 273 cases (85.3%) adults were present. 

In Table 1, all the retrieved foreign bodies are described. Children most frequently ingested coins (268 cases, 83.8%), that eventually remained stuck in the esophageal portion; among the recovered items, other round objects like buttons (11 cases, 3.44%) and plastic pieces (15 pieces, 4.69%) were subsequent for frequency. Most children showed vomiting (92, 28.7%). In 75 cases (23.4%), odynophagia was encountered. Lower frequencies were seen for sialorrhea (38 cases, 11.86%), ptyalism (31 cases, 9.69%), and dysphagia (28 cases, 8.75%). However, 47 cases (14.69%) showed no symptoms.

In 145 cases (45.31%), diagnosis was formulated within 3 hours after the injury and in 146 cases (45,6%) with a delay greater than 3 hours but not longer than 24 hours; only in 29 cases (9.06%), the FB was detected after more than 24 hours.

The FBs were located in the majority in the middle esophagus (269 cases, 84.1%).

Removal was performed in all cases, in 34 by esophagealforceps and in 286 cases using Magill hypopharyngeal forceps. General anaesthesia was administered in all cases. There was just one case where complications were observed (0.31%), presenting esophageal perforation.

In Table 2, a comparison between characteristics of the present case series and characteristics of previously published case series is provided. Some information (such as the adult presence) is frequently underreported, and more generally, information is not univocally categorized, impairing the possibility to perform any comparative effort. 

## 4. Discussion

FB injuries in the upper digestive tract continue to be a common health problem in paediatric patients. In the present study, the majority of cases involved children between 2 and 9 years of age, showing similarities with the results of the ESFBIs study and with Lin's study [8]. Less often, older children have risky behaviours, and this decrease of risk is as well ensured by the augmented size of the esophagus. 

In scientific literature, the most frequent foreign bodies result in fish bones, metal objects such as batteries and coins, and broken tooth fragments [11, 12]. Tissue response to a foreign body varies according to the composition of the FB and to any associated bacterial overinfection. Organic fragments cause a greater acute inflammation in comparison to pieces of metal, plastic, or bone. The relative inert nature of plastic materials allows the relatively quick response of patient upon removal of the foreign body, implying a milder tissue inflammation. Several authors have enlightened some differences between Asian and Western paediatric injuries due to foreign body ingestion, claiming that the specific type, food influences age distribution and nature of esophageal foreign body [2, 13]. In our study of coins were by far the most frequent foreign bodies ingested, followed by plastic pieces and buttons [7–9, 14]. Unlike Asian literature [13, 15], bones were not found with a high frequency.

Objects' characteristics such as shape, dimension, and consistency are important in order to determine the damage that might occur. Rimell and Stool [16] performed a retrospective study in which they examined the characteristics of objects that had caused serious aerodigestive tract (airway, cricopharyngeal, or esophageal) injuries, with the definition of serious being indicated by the need of operative removal or the occurrence of death due to choking, as reported from the Consumer Product Safety Commission (CPSC). Their results confirmed previous reports found in the medical literature, showing that the risk of injury or death posed by food, toy or toy part, or another object depends upon its size, shape, and consistency [17, 18]. 

The majority of swallowed foreign bodies pass harmlessly and spontaneously through the gastrointestinal tract (GIT) [8], but in case of lodgement or toxicity of the object, the FBs must be rapidly identified and removed. The most frequent lodgement site described in literature is the cricopharyngeus muscle [19, 20], while in our study the middle part of the esophagus was most frequent. Sharp items can lodge anywhere, and patients who have esophageal abnormalities such as tracheoesophageal fistulas are at risk of entrapment in atypical locations. Although most objects pass easily through the intestine, entrapment can occur at the pylorus, at the ligament of Treitz, and at the ileocecal valve [21]. 

Ingestions can vary in presentation from an asymptomatic state to respiratory distress or acute abdomen. Esophageal objects can cause a foreign body sensation, drooling, or respiratory distress due to tracheal compression, gagging, dysphonia, vomiting, and dysphagia, depending on the location and the nature of the FB [22]. Although a high percentage of children presented symptoms as vomiting and odynophagia, there were also a large percentage of asymptomaticcases. The delayed onset of symptoms is associated with an increased risk of complications: when presentation occurs after more than 24 hours, with the FB producing local inflammation in the mean time, it can potentially result in obstruction and mucosal and muscular erosion causing emesis, abdominal distension, or gastrointestinal bleeding. When the episode of the ingestion remains undetected, symptoms of chronic illness can develop, including signs as fever and weight loss. Although rare, perforating objects are potentially life threatening because they may provoke the formation of a fistula between the esophagus and the innominate artery thus ensuing catastrophic bleeding [23, 24]. Other complications associated with retained esophageal foreign bodies are tracheal compression and erosion through the mucosa, with FBs migration into adjacent structures, such as the respiratory tract or the aorta.

For all the gastrointestinal foreign bodies, the type of object, its location, and child's symptoms dictate treatment. In 2005, Waltzman et al. performed a randomized trial in children with coins lodged in the esophagus after their ingestion, comparing relatively immediate endoscopic removal to the choice of observation for a definite period of time [25] and retrieved a high frequency of spontaneous passages within 16 hours of observation. Based on a spontaneous passage rate of 25% to 30% and on the absence of complications, they stated that an 8- to 16-hour period of observation is an appropriate management in children with esophageal coins assuming that the child is asymptomatic, the ingestion is recent, and the child has no underlying esophageal or tracheal abnormality. In Waltzman's intent, this approach would have obviated the need for anaesthesia and endoscopy, with no increase in risk. In a subsequent paper, he suggested that in symptomatic patients with an esophageal coin, immediate removal via endoscopy is recommended whereas for asymptomatic patients with an esophageal coin, data supported an expectant management for a period of 12–24 hours [26]. In 2008's editorial, Conners compared endoscopical removal of esophageal coins and spontaneous passage [27]. He confirmed previous studies stating that the non interventional method can be preferred when there are no physical, functional, or postsurgical abnormalities, stressing the potential utility of low-tech solutions like a drink of water or a piece of bread when dealing with low-risk patients. 

Although most gastric objects pass without complication and can be observed in the outpatient setting, approximately 70% of esophageal objects remain entrapped, especially those in the upper or mid-esophagus [21]. In our series, we have observed just one case with complications, but that cannot be a reason not to mention the extreme hazardousness of the injury. Particularly, among the most dangerous ingested foreign bodies, batteries cover one of the leading places and must be mentioned when dealing with children's FB ingestion. The frequency of ingested button batteries is about 10 per million population per year, and one in every 1,000 battery ingestions causes serious injuries [28]. The incidence of button batteries ingestion has increased during the past several years [29]. Before 1983, there were only 6 cases of button battery ingestion in medical literature [30, 31]. In battery ingestion, the mechanism of injury occurs by four different means including direct corrosive action due to leakage, toxic effect due to absorption of substances, low voltage burns, and pressure necrosis [32–34]. Liquefaction necrosis and perforation can occur in 4 to 6 hours after a disk battery is lodged in the esophagus [35–37]. In the wide range of batteries, button batteries are those with peculiar characteristics that make them particularly dangerous for children, both from behavioural and anatomical points of view.

Prompt endoscopical intervention is the gold standard for all complicated or high-risk situations, with particular relevance to sharp and pointed foreign bodies, such as denture with protruding hooks, shaving blades, and open safety pins, which increase the danger of perforation. These objects extraction requires special attention and experience. As previously mentioned in our study, just one complication was observed, therefore removal techniques other than endoscopy were preferred. In planning the extraction, one of the important points to consider is the proper choice of the instruments. In our study, the greatest part of the extraction was performed through Magill forceps in general anaesthesia, and it showed a very low percentage of complications. The use of Magill forceps was seen in 286 cases. Magill forceps is a well-studied technique for the extraction of foreign bodies from the upper and medium part of the esophagus [38–40]. The use of esophageal forceps, performed in 34 cases, is preferred when dealing with distal foreign objects not requiring endoscopy.

As showed from all the studies here mentioned, the retrieved foreign bodies were objects usually available at home such as coins or plastic pieces, instead of toys or specific children's objects. That seems to reassure about the safety granted from the regulations imposed to toy's production, having the SPTF (Small Part Test Fixture) the most widely used test to define which objects might lead to injuries and which can be labelled as safe, as analyzed in the literature [6, 16]. Furthermore, the type of object causing injury was correlated with the age of the child injured as previously shown, following the proven strict linkage between injuries and children's development. Furthermore, the anatomy of gastrointestinal tract considerably changes in the first few years of life as maxillofacial structures extend forwardly and inferiorly and the larynx drops. These anatomical changes affect the risk associated with choking, aspiration, or ingestion of toys, toy parts, and FBs in general.

The clinical management of patients is effective in removing FBs and in reducing the impact of the accident. This is not directly impacting the rate of hospitalization, which is often seen as a precautionary measure, in particular complying to the age of the involved children. An expectant management for a period of 12–24 hours can be chosen when dealing with low-risk patients. 

Adult presence was recorded in the preponderance of cases, and the most common activity that the children were performing while they ingested the FBs was playing. Prevention of FB ingestion is not addressed adequately in families, both in terms of stressing the need of active supervision of children when playing, eating or interacting with objects inadequate to their age and not supposed to be within reach and also in informing about the necessity of a prompt intervention, since FBs ingestion is often not perceived as an accident requiring an urgent and specialized treatment.

The inadequacy of adult supervision has been largely reported [41, 42] and shows the importance of the implementation of education campaigns meant to properly estimate the overall risks decrease in preventing FBs ingestion.

In this context, doctors' role is fundamental in educating adults dealing with children, not only from a preventive point of view, but also in diminishing the impact that this kind of injuries has on Public Health. 

## Figures and Tables

**Table 1:** Description of retrieved foreign bodies.

Foreign Bodies	
Coins	268
Bones	15
Plastic pieces	15
Button	11
Metal	3
Tags	3
Pin	2
Ring	2
Toy	1
*Total*	320

**Table 2:** Comparison among characteristics recorded in the present case series and in 4 published case series. Data are given as percentage.

First author, publication year, country, number of retrieved FB					

	Buenos Aires 2009, Argentina, 320 FB	Gregori et al. 2010, [7] Europe, 186 FB	Little et al. 2006, [9], USA, 555 FB	Lin et al. 2007, [8], Taiwan, 38 FB	Van As et al. 2003, [10], South Africa, 735 FB
Distribution by sex					
Males	50.94	61	53.7	58.1	49
Females	49.06	49	46.3	41.9	51

Distribution by age					
Mean age	4.58	2	3.24	4	No available data. 3 years incidence peak (24)

FB type					
Organic FB	4.7	19.9	12	5.3	8
Inorganic FB	93.3	80.1	88	94.7	92

Adult present					
Yes	85.3	89.8			
No	14.7	10.2			

Location: Esophagus					
Upper	8.4		73	26	
Middle	84.1		14	5	
Distal	7.5		12	7	

Most frequent symptom	Vomiting (28.7)	Dysphagia (23)	Dysphagia (37)	Odynophagia (84)	

Most frequent complication	Esophageal perforation (0.31)	Pneumonia (1.08)	Epistaxis (0.36)	Erosive esophagitis (2.6)	No complications
